# Disturbance of phylogenetic layer-specific adaptation of human brain gene expression in Alzheimer's disease

**DOI:** 10.1038/s41598-021-99760-5

**Published:** 2021-10-12

**Authors:** Natasha Andressa Nogueira Jorge, Uwe Ueberham, Mara Knobloch, Peter F. Stadler, Jörg Fallmann, Thomas Arendt

**Affiliations:** 1 Bioinformatics Group, Department of Computer Science, Interdisciplinary Center for Bioinformatics, 04107 Leipzig, Germany; 2 Paul Flechsig Institute for Brain Research, University of Leipzig - Medical Faculty, Leipzig, Germany; 3 Max Planck Institute for Mathematics in the Science, Leipzig, Germany; 4 Institute for Theoretical Chemistry, University of Vienna, Wien, Austria; 5 Facultad de Ciencias, Universidad Nacional de Colombia, Bogotá, Colombia; 6 Santa Fe Institute, Santa Fe, USA

**Keywords:** Alzheimer's disease, Data processing, Gene ontology

## Abstract

Alzheimer's disease (AD) is a progressive neurodegenerative disorder with typical neuropathological hallmarks, such as neuritic plaques and neurofibrillary tangles, preferentially found at layers III and V. The distribution of both hallmarks provides the basis for the staging of AD, following a hierarchical pattern throughout the cerebral cortex. To unravel the background of this layer-specific vulnerability, we evaluated differential gene expression of *supragranular* and *infragranular layers* and *subcortical white matter* in both healthy controls and AD patients. We identified AD-associated layer-specific differences involving protein-coding and non-coding sequences, most of those present in the *subcortical white matter*, thus indicating a critical role for long axons and oligodendrocytes in AD pathomechanism. In addition, GO analysis identified networks containing synaptic vesicle transport, vesicle exocytosis and regulation of neurotransmitter levels. Numerous AD-associated layer-specifically expressed genes were previously reported to undergo layer-specific switches in recent hominid brain evolution between layers V and III, i.e., those layers that are most vulnerable to AD pathology. Against the background of our previous finding of accelerated evolution of AD-specific gene expression, here we suggest a critical role in AD pathomechanism for this phylogenetic layer-specific adaptation of gene expression, which is most prominently seen in the white matter compartment.

## Introduction

Alzheimer's disease (AD) is a progressive neurodegenerative disorder, neuropathologically characterised by fibrillar aggregates of the microtubule-associated protein tau and the amyloid β-peptide (Aβ), giving rise to the characteristic hallmarks of the disease in the form of neurofibrillary tangles and neuritic plaques, respectively. Accordingly, the presence of both tangles and plaques within the cerebral cortex provides the basis for post-mortem diagnosis^1^, as well as more recently, for in vivo diagnosis with PET tracers^2,3^. The formation of both tangles and plaques is likely a consequence of a pathological cascade involving the progressive conversion from oligomeric to fibrillar aggregates of tau and Aβ, resulting in a neuronal loss by a mechanism that still is largely unknown. This process of neurodegeneration might take years or even decades. It originates most probably in the subsynaptic compartment affecting long-axon neurons earlier and more severely. Structural impairments of long-axon neurons can, thus, be detected in imaging studies as white matter changes already many years before the onset of symptoms^4^.

While the exact mechanism of building up tangles and plaques is not yet understood very well, it has been firmly established that their progressive formation throughout different brain areas follows a defined hierarchical pattern that is robust enough to provide the basis for the neuropathological staging of the disease^5–7^. This development of AD pathology progressively involving an increasing number of areas likely follows the route of intracortical fibres, a process referred to as "spreading". However, even within cytoarchitectonically defined areas, the distribution of both plaques and tangles systematically follows some sub-area specific inhomogeneities; the exact nature of the latter is unknown^8^. Moreover, in addition to regional differences in vulnerability amongst cortical areas, tau and Aβ pathologies show a predilection for cortical layers.

The typical isocortex is formed by a six-layered structure characterised by specific cellular elements and connectivity patterns. Roughly, layers can be functionally divided into three parts. Layer IV is the main input layer, where major sensory afferentation of the cortex terminates at granule neurons, giving rise to the designation as the *internal granular layer*. Layers I to III, localised above layer IV, thus called *supragranular layers,* are the primary origin and termination of intracortical connections to either the same or the other hemisphere. The most prominent neurons are small layer-II/III pyramidal neurons, medium-sized layer-III pyramidal neurons, and inhibitory interneurons. The *infragranular layers* V and VI are the main origins of all principal long-ranging descending connections to subcortical areas. Large pyramidal neurons and inhibitory interneurons are the major neuronal components. Both input and long-ranging output fibres of the cortex club together below layer VI, forming the cortical white matter. In AD, neuritic plaques are preferentially localised to layers II and III, while neurofibrillary tangles are mainly found in pyramidal neurons of layers III and V^9^.

Given these differences in cellular components and connectivity patterns of *supra*- and *infragranular layers*, it would be challenging to identify those cellular components that account for different vulnerabilities. In the present study, however, we took an initial step towards identifying molecular networks shaping locally circumscribed vulnerability. Through a comparative transcriptome analysis of *supragranular* and *infragranular layers* together with a white matter analysis by high-throughput RNA sequencing in both healthy brain and AD, we aim to identify potential signatures of vulnerability together with molecular network components that are critically affected in AD.

## Results

The present study was performed on the temporal cortex of three healthy controls (HC) and three age-matched patients with AD. High throughput RNA-sequencing was performed on samples spanning the entire cortical depth, i.e., comprising all layers, including *subcortical white matter* (ALL), and samples where the *supragranular layers* (SUP), *infragranular layers* (INF) or the *subcortical white matter* (SWM) were individually dissected from surrounding tissue. After removing the adapter sequence and low-quality reads, an average of ~ 107,166,000 reads per sample was obtained (Supplementary Table S1).

### Layer-specific differences in gene expression within healthy controls and AD

To establish whether we could identify layer-specific differences in gene expression with our approach, we first analysed the differentially expressed genes (DEG) between SUP, INF and SWM separately for both HC and AD.

**Figure 1:**
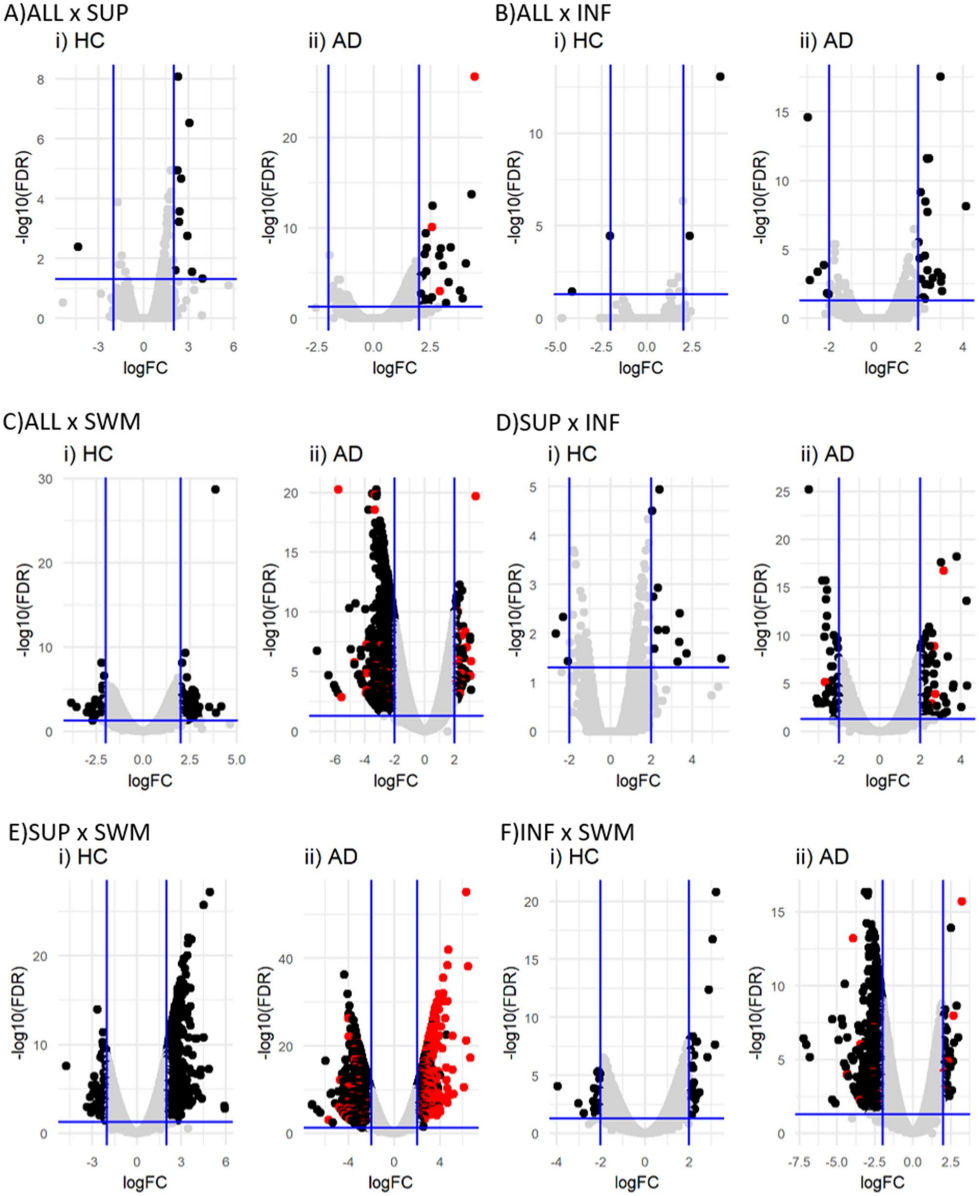
Differentially expressed genes amongst HC and AD layers. Volcano plot showing the DEG amongst the layers in the HC (i) and AD (ii) comparisons. All comparisons presented DEG in common (red dots in all panels), except for ALL and INF.

In HC, the number of DEG comparing SUP and INF was relatively small, amounting to a total of 15 (Table 1 and Fig. 1i), comprising protein-coding genes, lncRNAs and other non-protein-coding genes to about one third each. Expectedly, the amount of DEG was much higher between grey matter layers and SWM, amounting to a total of 634 and 62 comparing SUP with SWM and INF with SWM, respectively. In comparing INF with SWM, DEG classes comprise equal amounts of protein-coding genes, lncRNAs and other non-coding genes, while in the comparison between SUP and SWM, coding genes make up half of the total DEG.

**Table 1:** DEGs biotype amongst Healthy control and Alzheimer's disease.

Condition	HC			AD		
Comparison	SUP		INF	SUP		INF
	INF	SWM	SWM	INF	SWM	SWM
lncRNA	6	173	17	51 (1)	520 (143)	229 (6)
miRNA	0	7	0	2	14 (4)	5
Processed pseudogene	0	27	4	3	54 (20)	23 (1)
Protein coding	5	341	18	62 (3)	1415 (304)	470 (12)
snoRNA	2	38	10	0	24 (2)	15
snRNA	0	6	6	0	2	0
Transcribed unprocessed pseudogene	2	8	2	1 (1)	25 (6)	11
Unprocessed pseudogene	0	3	1	0	9 (3)	5
miscRNA	0	10	3	1	15 (5)	7
Transcribed processed pseudogene	0	2	0	0	15 (1)	5
TEC	0	14	0	1	37 (9)	16
Polymorphic pseudogenes	0	1	0	0	1	0
scaRNA	0	2	0	0	0	0
Unitary pseudogene	0	0	0	0	1	0
Total	15	634	62	121 (5)	2135 (497)	788 (19)

The number of DEG in common between the HC and AD comparisons are within parenthesis.

In AD, the expression pattern throughout the cortex was more heterogeneous than in HC, resulting in a higher number of DEG for all comparisons made (Table 1 and Fig. 1ii). 121 DEG were identified comparing SUP with INF, an eightfold increase, while 2.135 and 788 DEG could be detected comparing SUP with SWM and INF with SWM, respectively, corresponding to a threefold and 13-fold increase. The proportion between coding and non-coding DEG was also shifted, with about half to two-thirds of all DEG represented by protein-coding genes, which together with the higher overall number of differences reflects a drastic increase in the number of differentially expressed coding genes. Of note, many of the 62 protein-coding DEG identified in the SUP with INF comparison were already previously linked to AD or reported in a brain layer-specific context, such as, e.g., *AQP1*^10,11^, *CUX2*^12–14^, *HCN4*^14^, *PVALB*^13^, *RORB*^12,13,15^, *SLC17A6*^16^, *SYT2*^17^ or *TPH2*^18,19^ (Table 2). Moreover, our analysis also shows that the differential expression of protein-coding genes between SUP and INF is paralleled by significant differences between SUP and SWM expression, while differences between INF and SWM are less pronounced.

**Table 2:** Protein-coding brain layer associated DEG in AD in SUP versus INF layers.

Gene	Gene name	Comparisons				logFC^$^	Adj. *P*-value^$^
		SUP		INF			
		ALL	SWM	ALL	SWM		
*ADAMTS4*	ADAM metallopeptidase with thrombospondin type 1 motif 4	–	I	–	–	2.04	1.83E-12
*ADRA1D*	Adrenoceptor alpha 1D	–	D	I	–	− 2.66	2.02E-12
*APLNR*	Apelin receptor	I	I	–	I	3.79	6.85E-23
*AQP1*	Aquaporin 1	I	I	–	–	3.03	4.04E-22
*CD44*	CD44	I	I	–	–	2.43	7.71E-15
*CUX2*	Cut like homeobox 2	–	D	I	–	− 2.65	8.07E-18
*DUOX1*	Dual oxidase 1	I	I	–	I	2.37	5.00E-05
*HCN4*	Hyperpolarisation activated cyclic nucleotide-gated potassium channel 4	–	D	–	–	− 2.13	1.17E-05
*KLK6*	Kallikrein related peptidase 6	–	I	–	–	2.16	6.75E-13
*LRP2*	LDL receptor-related protein 2	–	I	–	–	2.06	2.66E-11
*NEFH*	Neurofilament heavy	–	I	–	–	− 2.13	4.81E-13
*NEUROD1*	Neuronal differentiation 1	–	I	I	–	− 2.11	5.44E-04
*PVALB*	Parvalbumin	–	D	–	–	− 2.23	4.58E-08
*RORB*	RAR related orphan receptor B	–	D	–	–	− 2.25	8.20E-14
*SLC17A6*	Solute carrier family 17 member 6	–	D	–		− 2.6	5.35E-16
*SNCG*	Synuclein gamma	–	D	–	–	− 2.07	2.53E-13
*SYT2*	Synaptotagmin 2	–	D	I	–	− 3.51	3.54E-30
*TDO2*	Tryptophan 2,3-dioxygenase	–	D	–	–	− 2.06	2.34E-04
*TNC*	Tenascin C	I	I	–	–	3.16	3.99E-21
*TPH2*	Tryptophan hydroxylase 2	–	D	–	–	− 2.29	1.95E-04
*VIPR2*	Vasoactive intestinal peptide receptor 2	–	I	–	–	− 2.02	3.61E-07

I: increased in the first term of the comparison; D: decreased in the first term of the comparison; -: unchanged. Underline genes were also repeatedly associated with AD. ^$^: values referent to the comparison between SUP and INF.

### Layer-specific differences in gene expression between healthy control and Alzheimer's disease

To better understand the physiological and AD-related pathophysiological characteristics of layer-specific gene expression, we next sought to compare the expression patterns between HC and AD. To first assess the overall magnitude of DEG between HC and AD, we started the comparison using samples comprising all cortical layers, including *subcortical white matter* (ALL).

Only 17 DEG between HC and AD were identified in ALL (Table 3). Out of these, 6 were downregulated and 11 up-regulated (Supplementary material Fig. SF1 and Supplementary Table S2). Amongst these DEG, the majority codes for proteins, while only 3 are annotated as pseudogenes, 1 lncRNA, 1 miRNA, and 1 transcript still to be confirmed (TEC). Thus, for all comparisons made, most DEGs comprise protein-coding genes, followed by lncRNAs (Table 3).

**Table 3:** Biotype of the DEG between HC and AD in each layer.

Biotype	Comparison			
	ALL	SUP	INF	SWM
lncRNA	1	6	3	44
miRNA	1	1	0	0
misc RNA	0	2	0	0
Processed pseudogene	1	1	0	5
Protein coding	11	12	12	66
scaRNA	0	1	0	0
snoRNA	0	2	0	3
snRNA	0	4	0	0
TEC	1	1	0	0
Transcribed processed pseudogene	0	0	0	2
Transcribed unprocessed pseudogene	1	1	1	1
Unprocessed pseudogene	1	4	1	4
Total	17	35	17	125

The only lncRNA found differentially expressed was *AC119673*, which is located antisense to the *PM20D1* gene, known as a quantitative trait locus (QTL) of AD and proposed as a potential blood-based biomarker for AD^20^. Among the protein-coding genes (Table 4), most were previously identified to be associated with AD, such as *PTGER3,* reported as an AD-associated hub gene^21^. SNPs in the *LIPG* gene are associated with AD and cardiovascular diseases^22^. In addition, *KIF25* was found hypermethylated in AD^23^, *HLA-DQB1* has alleles showing association to AD^24,25^, *CHRM5*, a cholinergic receptor, is up-regulated in AD^26^, *HLA-DRB5 was* identified as a risk gene for AD^25^, *MTRNR2L12* has been proposed as a candidate blood marker of early AD-Like Dementia in adults with Down Syndrome^27^, and *ADAMTS18* possesses secretase activity, a relevant feature in AD development/progression.

**Table 4:** DEG in ALL between HC and AD.

Gene name	Gene name	logFC	Adj. *P*-value
*PTGER3*	Prostaglandin E receptor 3	2.13	1.54E-05
*LIPG*	Lipase G, endothelial type	− 2.69	4.61E-02
*LTBP2*	Latent transforming growth factor beta binding protein 2	− 2.23	8.48E-03
*PTGIS*	Prostaglandin I2 synthase	− 2.20	3.96E-02
*KIF25*	Kinesin family member 25	− 2.27	9.06E-03
*ADAMTS18*	ADAM metallopeptidase with thrombospondin type 1 motif 18	− 2.37	8.48E-03
*COL3A1*	Collagen type III alpha 1 chain	− 2.28	3.79E-02
*HLA-DQB1*	Major histocompatibility complex, class II, DQ beta 1	− 2.64	3.93E-03
*CHRM5*	Cholinergic receptor muscarinic 5	− 2.59	5.50E-03
*HLA-DRB5*	Major histocompatibility complex, class II, DR beta 5	− 4.58	1.02E-05

Underlined genes were already reported in AD.

### Similarities and differences in layer-specific DEG in healthy control and Alzheimer's disease

To better characterise the specific effects of AD in each brain layer, we first identified those DEG that were found in both HC and AD comparisons (red dots in Fig. 1ii), a total of 521 DEG, with most of them coding for proteins. Out of all the DEG shared between comparisons, only the miRNA hsa-miR-3687 follows an opposite expression trend, being up-regulated in AD ALL compared with AD SUP (logFC: 2.92) but down-regulated in HC ALL when compared with HC SUP (logFC: − 4.35).

**Figure 2:**
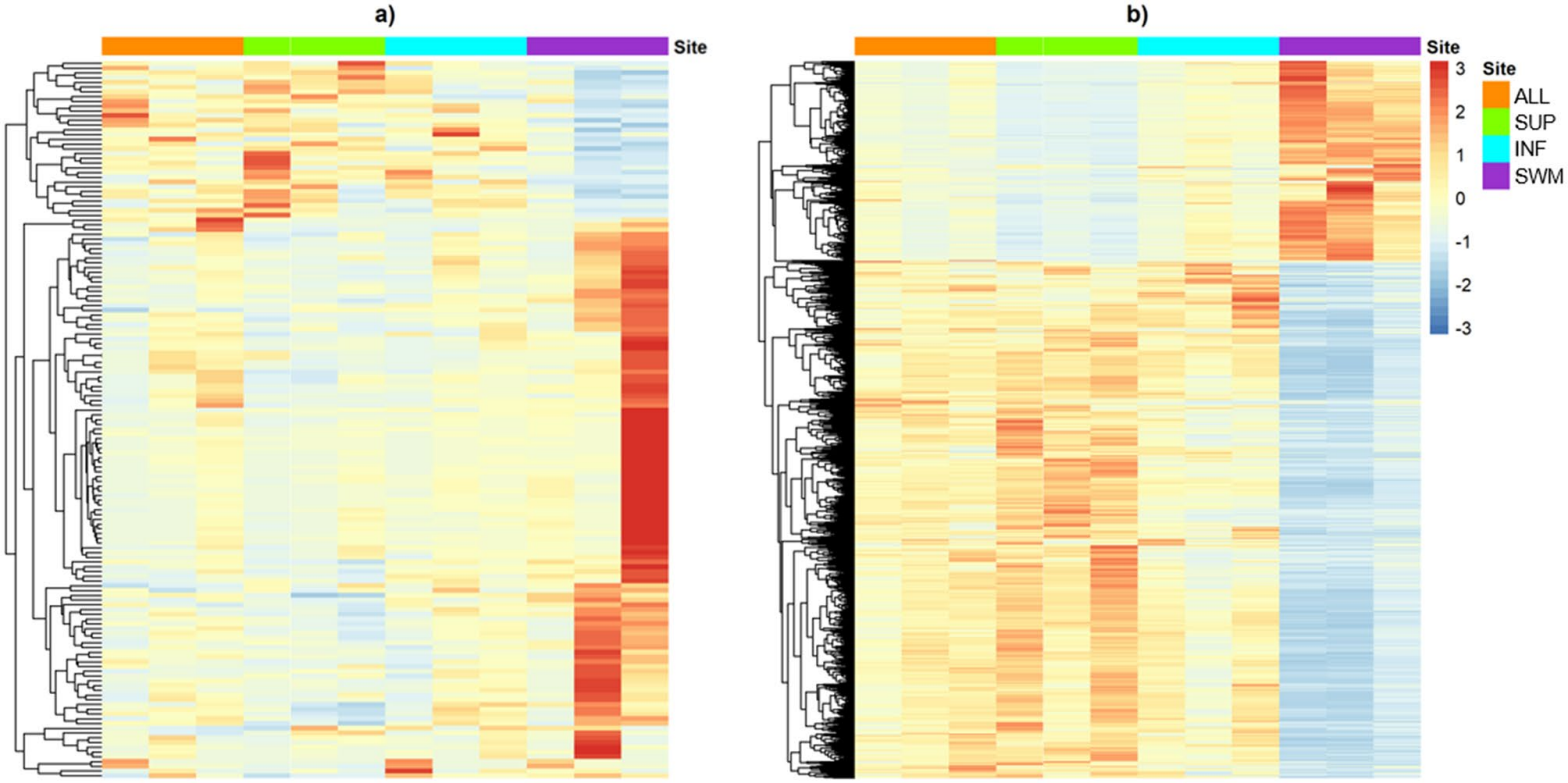
Unique and common DEG between AD and HC. Heatmaps showing the distinct expression profile of the DEG found uniquely in the HC (**a**) or AD (**b**) comparisons.

Despite the significant number of DEG in common, both HC and AD conditions still present distinct expression profiles when considering unique DEG (Fig. 2 a and b). While most of the unique DEG are up-regulated in the HC brain SWM compared to ALL and the layers (SUP, INF) (Supplementary Tables S5, S7, and S8), in AD, the opposite holds, where DEG are mostly down-regulated in SWM compared to ALL and layers (SUP, INF) (Supplementary Tables S11, S13, and S14). Consolidated, the SWM layer of both HC and AD comprise more distinct profiles than SUP and INF.

### Layer-specific biological pathways within healthy controls and AD

To identify physiological and AD-related features of the layer-specific expression pattern, we next performed a Gene set enrichment analysis (GSEA) for each comparison made.

Concerning HC, only those comparisons that involved SWM showed significant GO terms (Supplementary Tables S20 to S22). Here, we identified several Biological Processes (BPs) related to synaptic function, such as "signal release", "cell–cell signalling", "regulation of action potential", and brain functions such as "cognition" and "behaviour". Also, the comparisons involving the INF were related to BPs involved in secretion, such as "neurotransmitter secretion", "hormone secretion", "peptide secretion", and exocytosis, besides "learning" and "memory".

**Figure 3:**
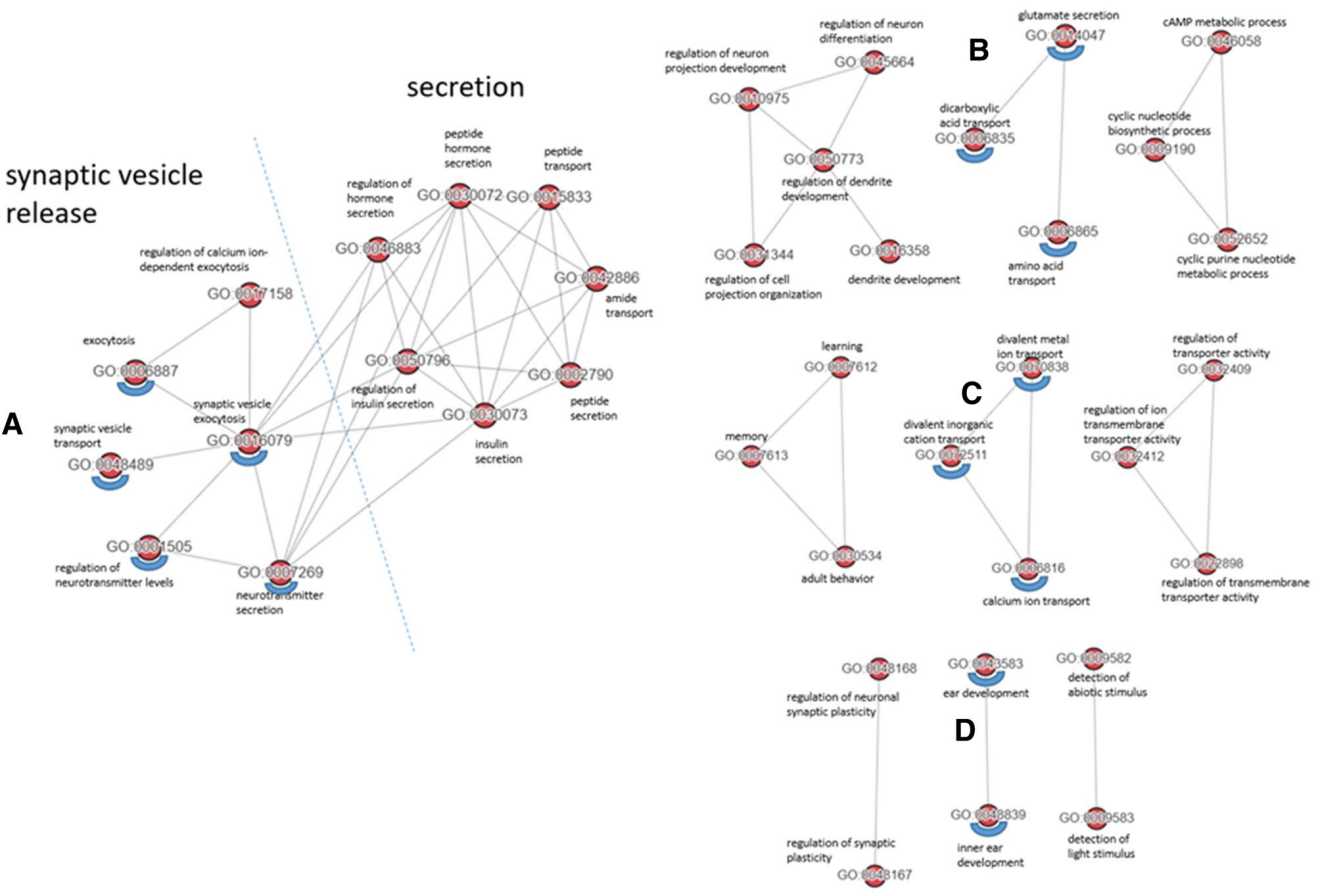
Unique meaningful GO clusters specific for AD brain. The GO terms underlined with blue semicircles were found in each comparison.

All those GO-terms found in HC could be replicated in AD, but additional BPs could be identified (Supplementary Tables S15 to S19 and Supplementary Tables S25 to S27). Four AD-specific GO clusters reproducibly present in all comparisons made (Supplementary Tables S25 to S27) were identified applying NaviGO^28^ and Resnik's similarity approach^29^ (Fig. 3). Presynaptic transport processes were clustered most prominently, with 'synaptic vesicle release' representing the most prominent cluster (Fig. 3A), which is in line with a common hypothesis that synaptic dysfunction is the major correlate of cognitive decline^30^, precedes neuronal cell death and is already detectable in pre-symptomatic stages of the disease^31,32^. Of note, recent data underline the early role of presynaptic vulnerability^33^ in contrast to postsynaptic compartments. In addition, 'inner ear development'^34^ was also detected as a cluster (Fig. 3D), supporting the growing evidence that several genes affecting proper inner ear development, e.g. alpha-synuclein (*SNCA*)^35^ or beta-secretase (*BACE1*)^36^, are also involved in neurodegenerative disorders including AD. This could reinforce the proposal of hearing loss as an interplay of 'peripheral' and 'central' hearing dysfunction, which is linked to cognitive decline and has been attributed increasingly a modifiable risk factor for AD^37^.

"Blood circulation" and "Circulatory system process" were found significant in four out of the six comparisons (Supplementary Table S23).

The most prominent GOs were identified in AD comparisons that involved SWM, amounting to an overlap of 79 terms, which comprised "synaptic vesicle exocytosis", "regulation of dendrite development", "sensory perception", "neurotransmitter secretion", "synaptic vesicle transport", "regulation of neuronal synaptic plasticity", "regulation of neurotransmitter levels", "neuron-neuron synaptic transmission", "membrane depolarisation", "regulation of neuronal synaptic plasticity", and "adult behavior". This clearly points towards a substantial synaptic dysfunction as a critical early event in AD as suggested previously^31,32^. Further, the term "demyelination" exclusively found in AD comparisons, was an additional indicator of fibre degeneration in AD^4^.

Next, we evaluated the expression patterns of the unique DEG in the most significant BP for each comparison. Although some pathways are present in more than one comparison, such as "extracellular matrix organisation" and "extracellular structure organisation" in both SUP with ALL and INF with ALL comparisons, they are represented by different genes, such as *SFRP2* (in SUP with ALL) and *MYH11, COL5A2,* and *COL8A1* (in INF with ALL), all up regulated in the ALL samples when compared to the others. This is also consistent with the cellular environment alterations caused by the accumulation of β-amyloid^38^.

The comparisons involving the SWM samples all show BP involved in signal transmission, such as "signal release", "regulation of synaptic transmission", and "generation of a signal involved in cell–cell signalling. Again, most of the DE genes in significant BP are downregulated. Interestingly, exceptions are the genes *KLK6* and *TNC,* which are both up-regulated in SWM in the ALL with SWM comparison. The *KLK6* gene is part of the "regulation of nervous system" pathway, while the *TNC* is part of the "neuron projection morphogenesis" and "axonogenesis". *KLKL6* is suggested as a microglia marker^39^, and *TNC* is an extracellular protein involved in developing and repairing neural tissues^40^. In the SUP with SWM comparisons, the genes *CD38*, *GAB2, NGFR, NTN1, PSEN1, P2RX7, PRAM1,* and *SEMA4D* are all up-regulated in SWM and part of BP such as "exocytosis", "axon guidance", "learning memory", and "regulation of synaptic activity". Lastly, in the INF with SWM comparison, the gene *STC2* is up-regulated in SWM and part of the "generation of a signal involved in cell-to-cell signalling" and "cation transport" BPs. These findings underscore the importance of SWM as a most critical compartment in AD pathogenesis.

## Discussion

Brain regional vulnerability has been the subject of previous investigations^8,41–44^. Some early studies examined gene expression in selected human neocortical laminae using microarrays to address aspects such as the cortico-cortical network architecture^45^, schizophrenia^46,47^ or frontotemporal lobar degeneration^48^. More recent studies used RNA-sequencing to compare gene expression in other primates^49^ and single-nucleus RNA-sequencing to obtain layer-enriched expression^50^. However, to the best of our knowledge, the aspect of layer-specific vulnerability has not been addressed for AD at the molecular level using RNA-sequencing, so far^45–50^.

Our present intention to take a layer-specific approach has also been stimulated by recent functional cognitive brain imaging developments allowing for ultra-high structural resolution, demonstrating layer-specific activity patterns in mental events^43^. Thus, with the present study, we hope to add a new molecular dimension to those differences that have already been established between *supra*- and *infragranular cortical layers* in AD concerning cytoarchitectonics, connectomics and vulnerability.

The comparison of gene expression between the temporal cortex of HC and AD on samples comprising all brain layers and *subcortical white matter* (ALL) allowed to detect a small number of DEG, with most of them already being reported concerning AD. Since the ALL-samples comprise different cell types such as neurons, including their processes, astrocytes and oligodendrocytes, only the most prominent differences beyond cell type and spatial location might be expected to show up.

In contrast, all comparisons between different layers, including SWM, revealed layer-specific DEG in the HC brain, which were even more frequent in the corresponding AD brain comparisons. Protein coding genes were found differentially expressed most often, followed by ncRNAs, with both together constituting a fraction of more than 55% and 88% of all DEG in HC and AD, respectively. Of note, the number of both coding and non-coding RNAs found differentially expressed is at least more than three times increased in the corresponding AD comparisons. This adds further evidence to a critical role of non-coding RNAs in AD pathomechanism, which remains an understudied aspect of the disease. Along this line, we recently could show that AD-associated protein-coding RNA but even more so, non-coding RNAs show accelerated evolution^51^, indicating a link between most recent hominid brain evolution and vulnerability towards AD. This aspect might well be linked to the process of phylogenetic development of a six-layered cortical structure.

### Specific synapse-related GO terms are uniquely affected in AD

For HC, significant layer-specific GO terms were found for comparisons involving SWM, which are often related to synaptic function, including regulation of action potentials and signal release. In AD, additional GOs were identified; they form a network containing "synaptic vesicle transport", "synaptic vesicle exocytosis", and "regulation of neurotransmitter levels". In addition, the GO term "glutamate secretion" was represented prominently, which against the background of the critical involvement of *subcortical white matter*, as documented in the present study, and in agreement with previous findings, clearly identifies presynaptic changes of long axons, mainly glutamatergic neurons, as most early and most constant finding in AD pathomechanism^31,52,53^.

### *Subcortical white matter* as a critical compartment in AD pathogenesis

For all comparisons made, most DEG were found in comparisons that involved SWM. While in HC, this might simply reflect genuine differences in cellular composition between grey and white matter, it indicates a prominent role for the subcortical fibre compartment together with associated cellular elements such as oligodendrocytes in AD pathology.

While white matter changes have not been in the direct focus of AD research until recently, it becomes clearer that they represent an earliest and most critical aspect of the disease^44^ that correlates very well with clinical/cognitive traits of the disease^54^. Still, the phenotypic link between the progression of neurofibrillary changes in AD and the myelination patterns, suggesting some dysfunction in oligodendrocytes as a potential cause or early trigger of AD, had been reported by Braak and Braak quite some time ago^55^. A recent study examining AD cases without co-morbitities also points to an early environment of altered oligodendrocytes into AD pathology^56^.

Our present findings in the changes in RNA expression pattern, mainly associated with a reduction of transcript expression in SWM, might well be seen against the background of disease-associated changes in axonal transport systems and its potential involvement in the spreading of tau pathology, which, according to recent findings by Braak and Tredici^57^, takes a cortico-cortical top-down route. Moreover, it is in line with the recent result of relatively low white matter integrity in subjective cognitive decline (SCD), which is assumed as one of the earliest stages on the continuum towards AD^58^.

Our recent observation on accelerated evolution of AD-associated genes^51^ and the recent findings of noticeable changes in the *subcortical white matter* is in direct agreement with a report on an about two-fold higher evolutionary rate of human white matter genes compared to pyramidal cell layers^49^. Human-specific organisation of layer-specific gene expression patterns might thus play an important role in the pathological process of AD development.

He and collaborators^49^ and Zheng and collaborators^59^ show that recent hominid brain evolution is associated with brain layer-specific switches in gene expression when comparing humans to chimpanzees. Two genes (*ULBP2*, *MGAT5*) out of the 18 reported by He and collaborators. (2017) that undergo a human-specific expression transition from layer V to layer III were detected in our AD specific DEG analysis. Furthermore, another five genes (*CHRNB3*, *CNTNAP4*, *AQP1*, and *NGB*), reported by this group as human brain layer-specific, were also found differentially expressed in our AD comparisons. In the work of Zhen and collaborators^59^, four out of the seven genes (*CRY*, *PRSS12*, *SCN4B*, and *SYT2*) show changes in expression patterns between layer V and layer III, when comparing mouse to human brain, were also detected amongst our AD-associated DEG. As layers III and V are the layers most typically involved in neurofibrillary degeneration, our data suggest a link between the layer-specific gene expression and its phylogenetic switch and AD pathology.

Though we did not find any differential expression of *MAPT* (microtubule associated protein tau) or *APP* (amyloid beta precursor protein), which gene products contribute to neurofibrillary tangles and Aβ plaque deposition in cortical AD layers, our data provide indications of layer specific modified expression of genes potentially affecting APP metabolism and Aβ generation. *ADAMTS4* was increased in SUP in AD brain compared to INF as well as to SWM (Table 4). This gene codes for a disintegrin-like and metalloproteinase with trombospondin type 1 motif and can cleave Aβ peptide sequence between Glu-3 and Phe-4^60^. Coexpression of *ADAMTS4* and *APP* resulted in HEK293 cells in the secretion of Aβ_4-40_ peptides^60^ supporting a contribution of this enzyme to the Aβ amyloid pathology in AD. Their elevation in SUP is paralleled by the most early formation of plaques compared to INF^9^. Moreover, the observed reduction of cortexin (*CTXN3*) in SUP of AD compared to INF (supplementary Table S12) could also contribute to the increased Aβ generation^61^ in this AD brain layer. This data are well in agreement with the hypothesis, that layer specific alterations of gene expression contribute to specific AD pathology.

Evolutionary alteration of layer specific gene structure and expression might affect neuronal plasticity and allow for a higher degree of cellular individuality, resulting in a remodelling of layer specific function, paving the way to a wider spectrum of cognitive abilities, potentially at the expense of increased vulnerability.

This process might be closely linked to a shift or redefinition of neuronal identity, since several coding genes, differentially expressed between SUP and INF in AD (Table 2) define specific cell types. For example, *PVALB* is coding for parvalbumin, a marker for a subset of interneurons, *TPH2* is coding for tryptophan hydroxylase, a marker for serotonergic neurons^62^, while *RORB* is known as a developmental driver of neuronal subtype identity in the neocortex^13,63^. Finally, alterations of *CUX2* expression (Table 2), a SUP layer specific marker^64^, might be linked to neurodevelopmental disorders such as autism and schizophrenia^65^, or epileptogenesis^64^.

We used layer-specific RNA sequencing to understand better molecular correlations of systematic differences towards AD-specific vulnerabilities in this work. Our findings indicate the importance of non-coding genes in layer-specific physiology of the human brain and AD-pathology. Furthermore, the DEG found corroborate the findings of pre-symptomatic molecular alterations in white matter that might be intrinsic to AD's origin. Lastly, many AD-associated layer-specifically expressed genes were previously reported to undergo layer-specific switches in recent hominid brain evolution, thus suggesting an evolutionary pattern critical to the genesis and development of AD.

## Methods

### Human brain tissue

Brain tissue of 3 AD patients and 3 healthy controls (Table 5) dying without any history of neurological or psychiatric illness was provided by the Brain Banking Centre Leipzig of the German Brain Net (GZ 01GI9999-01GI0299), operated by Paul Flechsig Institute of Brain Research (Approval # 282- 02). The diagnosis of AD was made on the basis of both clinical and neuropathological evidence according to the criteria of the International Working Group (IWG) for New Research Criteria for the diagnosis of AD^66^ in the revision of 2014 (IWG-2)^67,68^, the NIA-AA diagnostic criteria in the revision of 2011^69–71^, and the NIA-AA guidelines for the neuropathological assessment of AD^55,56^. Only cases with typical AD according to IWG-2 criteria were included. All cases were neuropathologically assessed for neurofibrillary tangle stage according to Braak and Braak^5^ and Braak et al.^72^, for Aβ/amyloid plaque score according to Thal et al.^7^ and for neuritic plaque score according to CERAD^1^. Neurofibrillary tangles and Aβ/amyloid plaques were detected by immunocytochemical labelling of phospho-tau (anti-human PHF-tau monoclonal antibody AT8; Thermo Scientific) and Aβ (beta-amyloid monoclonal antibody, 6E10; BioLegend), respectively. The severity of AD pathology was scored following the consensus guidelines for the neuropathologic evaluation of AD according to Hyman et al.^17^ and Montine et al.^73^. Case recruitment, autopsy and data handling have been performed in accordance with the ethical standards as laid down in the 1964 Declaration of Helsinki and its later amendments as well as with the convention of the Council of Europe on Human Rights and Biomedicine and had been approved by the responsible Ethics Committee of Leipzig University.

**Table 5:** Cases used in the study.

Case ID	Age	Sex	ABC scoring and neuropathological level of AD^1,5,7,72,73^				CDR^3,82–84^	Group
			A	B	C	Level		
310/20	80	F	0	0	0	not	0	control
445/25	83	M	0	1	0	not	0	control
175/25	85	F	0	0	0	not	0	control
1100/20	77	F	2	3	3	intermediate	1	AD
1300/25	82	F	2	2	1	intermediate	1	AD
25/20	76	M	3	3	3	high	1	AD

CDR: Clinical Dementia Rating. F: Female. M: Male. AD: Alzheimer’s disease.

### Tissue microdissection, RNA Isolation and RNA-sequencing

Deeply frozen tissue (stored at − 80 °C) of the temporal cortex (Brodman area 22) was cut in 45 µm sections in a standard cryostat at − 20 °C using Tissue-Tek O.C.T. Compound (Sakura Finetek Europe, Netherlands). Sections were mounted either for microdissection on MembranSlides 1.0 PEN (Carl Zeiss, Germany), which had been pretreated with UV-light for 30 min, or on SuperFrostPlus slides (Thermo Scientific, Germany) for Nissl staining which was used as reference to specific regions of interest. For Nissl staining sections on slides were dried (30 min) and subsequently incubated in ethanol solutions as indicated: 70% (1 min), 85% (1 min), 95% (30 min), 85% (30 s), 70% (30 s) and 50% (30 s). After staining with 1% cresyl violet in 50% ethanol for 2 min, sections were differentiated by incubation in increasing ethanol solutions (70%, 85%, 95%, 100%) followed by isopropanol treatment for 1 min and then cover.slipped with Entellan (Merck, Germany). Ethanol solutions for PEN slides were always prepared with DEPC-treated H_2_O. Slices on PEN slides were immediately fixated in pre-cooled 70% ethanol for 2 min, followed by dehydration in increasing ethanol solutions (85%, 95%, 100%; for 1 min each) and final drying for 2 min. Microdissection of indicated areas (SUP, INF, SWM) was performed using the Zeiss P.A.L.M. microbeam microscope system. Captured tissue was collected using micro-tweezers and transferred to RTL-buffer (RNeasy micro kit, Qiagen, Germany) for lysis. Total RNA was extracted using TRIzol reagent (Life technologies, UK). RNA quality assessment was performed using Agilent 2100 Bioanalyzer (RNA Pico chips; Agilent Technologies, USA) and only samples with RQN > 4.5 were further included. After treatment with DNaseI (NEB, Germany) RNA was purified using PCI reagent (Carl Roth GmbH, Germany). For depletion of ribosomal RNA *RiboMinus Eukaryote System v2* (Life Technologies GmbH, Germany) and for library preparation NEBNext Ultra II Directional RNA Library Prep Kit for Illumina (NEB, Germany) were used. RNA sequencing was performed on an Illumina HiScan SQ system.

### Bioinformatics sample pre-processing

The adapter sequence and reads presenting sequencing quality lower than 20 were removed using TrimGalore! version 0.6.3^74^ (https://www.bioinformatics.babraham.ac.uk/projects/trim_galore/). All comparisons were performed in R 4.0.0. 0n statistical environment, and all plots were done using the R packages ComplexUpset version 0.5.17^75^ (https://github.com/krassowski/complex-upset) and ggplot2 version 3.3.2^76^ (https://cran.r-project.org/web/packages/ggplot2/index.html).

### Differential gene expression

Trimmed and filtered reads were aligned against the GRCh38 version of the human genome (downloaded from Ensembl, release 98 on August 2019, http://ftp.ensembl.org/pub/release-98/fasta/homo_sapiens/dna/) using Star version 2.71a^77^ (https://github.com/alexdobin/STAR). Gene raw expression profile was obtained using the uniquely aligned reads, the featureCounts method from the subread version 2.0.1^78^ (https://bioconductor.org/packages/release/bioc/html/Rsubread.html) packages and the ENSEMBL gene annotation 98^79^. Differentially expressed genes were identified with EdgeR version 3.28.0^80^ (https://bioconductor.org/packages/release/bioc/html/edgeR.html) filtered for FDR ≤ 0.05 and |logFC|≥ 2. GO Biological processes enriched in this comparison were evaluated using the R package GAGE version 2.38.3^81^ (https://bioconductor.org/packages/release/bioc/html/gage.html) and filtered for q.val ≤ 0.05.

## Supplementary Information

Supplementary Information 1.

Supplementary Information 2.

## Data Availability

All sequencing data is available in the BioProject database under the BioProject ID: PRJNA752983 (http://www.ncbi.nlm.nih.gov/bioproject/752983).
