# Author Correction: Disturbance of phylogenetic layer-specific adaptation of human brain gene expression in Alzheimer's disease

**DOI:** 10.1038/s41598-021-02014-7

**Published:** 2021-11-15

**Authors:** Natasha Andressa Nogueira Jorge, Uwe Ueberham, Mara Knobloch, Peter F. Stadler, Jörg Fallmann, Thomas Arendt

**Affiliations:** 1 Bioinformatics Group, Department of Computer Science, Interdisciplinary Center for Bioinformatics, 04107 Leipzig, Germany; 2 Paul Flechsig Institute for Brain Research, University of Leipzig - Medical Faculty, Leipzig, Germany; 3 Max Planck Institute for Mathematics in the Science, Leipzig, Germany; 4 Institute for Theoretical Chemistry, University of Vienna, Wien, Austria; 5 Facultad de Ciencias, Universidad Nacional de Colombia, Bogotá, Colombia; 6 Santa Fe Institute, Santa Fe, USA

Correction to: *Scientific Reports* 10.1038/s41598-021-99760-5, published online 12 October 2021

The original version of this Article contained a formatting error in Table 2 where the Genes *ADAMTS4*, *ADRA1D*, *APLNR*, *AQP1*, *CD44*, *DUOX1*, *KLK6*, *LRP2*, *NEFH*, *NEUROD1*, *PVALB*, *SNCG*, *SYT2*, *TDO2*, and *TNC* were not underlined.

In addition, in Table 4, the Gene names *LIPG*, *PTGIS*, *ADAMST18*, *HLA-DQB1*, and *HLA-DRB5* were not underlined.

The original version of the Article has been corrected.

