# Supplementary material for: Disturbance of phylogenetic layer-specific adaptation of human brain gene expression in Alzheimer's disease
Source: Sci Rep. 2021 Oct 12;11:20200. doi: 10.1038/s41598-021-99760-5 (PMC8511061; doi:10.1038/s41598-021-99760-5)
Supplement: Supplementary file 1 — Supplementary Information 1. [file 41598_2021_99760_MOESM1_ESM.docx]

**Disturbance of phylogenetic layer-specific adaptation of human brain gene expression in Alzheimer's disease**

**Natasha Andressa Nogueira Jorge^1§*^, Uwe Ueberham^2§^, Mara Knobloch^2^, Peter F. Stadler^1,3-6^, Jörg Fallmann^1#^, Thomas Arendt^2#^**

^1.^ Bioinformatics Group, Department of Computer Science, and Interdisciplinary Center for Bioinformatics, Leipzig, D-04107, Germany.

^2.^ Paul Flechsig Institute for Brain Research, University of Leipzig - Medical Faculty, Leipzig, Germany

^3.^ Max Planck Institute for Mathematics in the Science, Leipzig, Germany

^4.^ Institute for Theoretical Chemistry, University of Vienna, Wien, Austria

^5.^ Facultad de Ciencias, Universidad Nacional de Colombia, Bogotá, Colombia

^6.^ Santa Fe Institute, Santa Fe, USA

*corresponding author


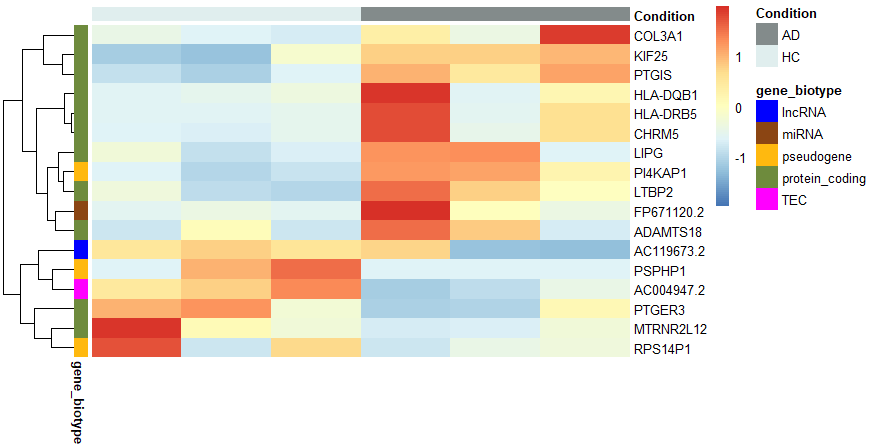


Supplementary Figure SF1; Heatmap of the differentially expressed genes (DEGs) between the Whole Brain control (HC) and Alzheimer’s (AD) samples. Totally, we found 17 DEGs, the majority encodes for proteins followed by pseudogenes.
